# Concurrent Hemophagocytic Lymphohistiocytosis and Thrombotic Microangiopathy Induced by Cytomegalovirus and Epstein-Barr Virus Co-infection in an Immunocompetent Young Adult: A Case Report

**DOI:** 10.7759/cureus.92654

**Published:** 2025-09-18

**Authors:** Maha El Amani, Firdaous Belkaid, Soukaina Haidouri, Tarek Dendane, Khalid Abidi

**Affiliations:** 1 Department of Clinical Hematology, Ibn Sina Hospital, Mohammed V University, Rabat, MAR; 2 Department of Critical Care Medicine, Ibn Sina Hospital, Mohammed V University, Rabat, MAR

**Keywords:** cytomegalovirus (cmv), epstein barr virus (ebv), hemophagocytic lymphohistiocytosis (hlh), immunocompetent adult, microangiopathic hemolytic anemia, thrombotic microangiopathy

## Abstract

Hemophagocytic lymphohistiocytosis (HLH) and thrombotic microangiopathy (TMA) are rare and life-threatening conditions that can be triggered by viral infections, though their simultaneous occurrence remains exceptional, particularly in immunocompetent individuals.

We report the case of a previously healthy 19-year-old woman who developed severe anemia, thrombocytopenia, and fever, rapidly progressing to status epilepticus and multiorgan failure. Laboratory findings were consistent with microangiopathic hemolytic anemia (MAHA), with a normal ADAMTS13 activity, and brain imaging demonstrated diffuse cerebral vasculitis with microthrombi. Extensive workup excluded autoimmune, malignant, toxic, and other infectious causes, while polymerase chain reaction (PCR) testing revealed significant cytomegalovirus (CMV) and Epstein-Barr virus (EBV) viremia. In parallel, the patient fulfilled six of the eight HLH-2004 criteria, including persistent fever, bi-cytopenia, hepatosplenomegaly, hyperferritinemia, hypertriglyceridemia, and hemophagocytosis on bone marrow examination, with an HScore of 259 (>99% probability of HLH), confirming the diagnosis of secondary HLH. Treatment included corticosteroids, ganciclovir, rituximab, cyclophosphamide, anticoagulation, and supportive care, resulting in gradual clinical improvement and full recovery. This case illustrates the pathogenic synergy between CMV and EBV in precipitating both HLH and TMA in an immunocompetent host, and highlights the importance of early diagnosis and multidisciplinary management in such rare and aggressive presentations.

## Introduction

Hemophagocytic lymphohistiocytosis (HLH) is a rare but severe hyperinflammatory syndrome characterized by excessive immune activation leading to multiorgan failure. It is classified into primary (genetic) forms, typically affecting children, and secondary forms, associated with viral, autoimmune, or neoplastic triggers [1].

Thrombotic microangiopathies (TMAs) comprise a heterogeneous group of disorders defined by microvascular thrombosis, resulting in microangiopathic hemolytic anemia (MAHA), thrombocytopenia, and organ ischemia. TMAs may be primary or secondary, the latter arising in the context of infections, malignancies, autoimmune conditions, pregnancy, malignant hypertension, or exposure to certain drugs or toxins [2].

Although infections are recognized triggers for both HLH and TMA - particularly in immunocompromised patients [3,4] - their concurrent manifestation in immunocompetent individuals is exceedingly rare.

We present a unique case of EBV/CMV (Epstein-Barr virus/cytomegalovirus) co-infection inducing simultaneous HLH and TMA in a previously healthy young adult.

## Case presentation

A previously healthy 19-year-old woman, with no known comorbidities or medication history, was admitted to the Clinical Hematology Department for evaluation of newly discovered anemia, associated with a persisting fever lasting for one week and peaking at 39°C. The patient denied any recent travel, exposure to toxins, or drug consumption. There was no personal or family history of immunodeficiency, hematological, or autoimmune disorders. 

A few hours following her hospitalization, the patient developed a generalized tonic-clonic seizure that rapidly progressed to status epilepticus. She was urgently transferred to the Intensive Care Unit (ICU) with a Glasgow Coma Scale (GCS) score of 3/15, necessitating endotracheal intubation and mechanical ventilation. Physical examination revealed cutaneous and mucosal icterus, mild splenomegaly, and papulonodular reticulate skin lesions on her back and bilateral lower limbs. There was no evidence of lymphadenopathy, mucosal bleeding, or petechiae. 

Initial laboratory work-up showed normocytic anemia, marked thrombocytopenia, elevated lactate dehydrogenase (LDH), indirect hyperbilirubinemia, and undetectable haptoglobin, suggestive of intravascular hemolysis. A direct antiglobulin (Coombs) test was negative. Reticulocyte count was elevated, and the peripheral blood smear revealed schistocytes accounting for 20% of red cells, consistent with MAHA. Further labs demonstrated acute renal and hepatic failure, hyperferritinemia, and elevated triglyceride levels (Table 1). Despite severe thrombocytopenia, coagulation parameters remained normal, and no disseminated intravascular coagulation was noted. 

**Table 1:** Results of the various biological parameters measured and their evolution over the 15-day hospitalization in the Intensive Care Unit. AST: Aspartate Aminotransferase; ALT: Alanine Aminotransferase; LDH: Lactate Dehydrogenase; IU: International Unit; Mg: Milligram

Serum test	Reference range	Day 1	Day 5	Day 10	Day 15
Hemoglobin (g/dL)	12.0-15.0	5.3	8.1	10.5	11.3
Leukocytes (× 10⁹/L)	4.0-10.0	12.6	15.3	12.1	11.8
Platelets (× 10⁹/L)	150-400	15	43	65	82
Reticulocytes (× 10⁹/L)	25-100	180	191	182	153
Schistocytes (%)	<1	20	11	<1	<1
C-reactive protein (mg/L)	<0.50	70	51	31.4	29.2
Ferritin (ng/mL)	30-340	9421	5021	2316	1320
Lactate dehydrogenase (IU/L)	125-220	350	340	270	250
Haptoglobin (g/L)	0.4-2.0	<0.1	<0.1	0.5	0.5
AST (IU/L)	6.0-30	203	101	66	34
ALT (U/L)	6.0-35	125	32	13	11
Total bilirubin (mg/L)	3-17	42	31	15	5
Indirect bilirubin (mg/L)	Total bilirubin - Direct bilirubin	36	25	9	2
Triglycerides (g/L)	0.3-1.5	3.1	2.1	1.1	0.9
Creatinine (mg/L)	6.0-11	26	21	15	4.9

**Figure 1:**
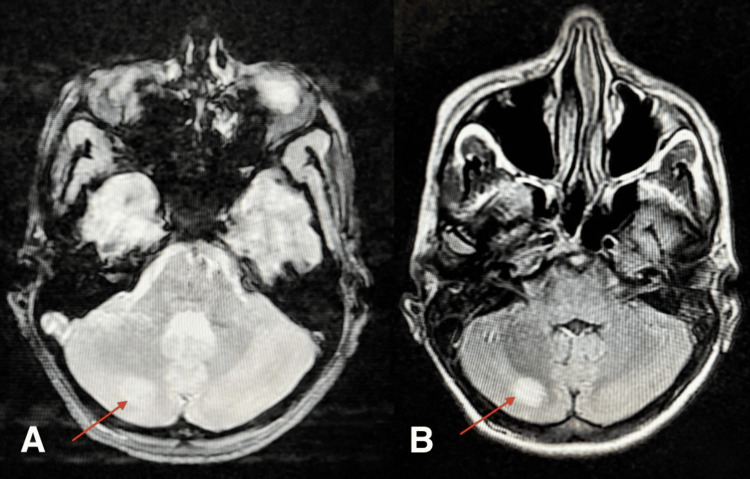
Axial brain MRI sequence of the patient, showing right cerebellar ischemic lesion. (A) T2-FLAIR sequence showing the same cerebellar lesion (red arrow). (B) T2-weighted sequence showing an acute ischemic lesion in the right cerebellar hemisphere (red arrow). MRI: Magnetic Resonance Imaging; FLAIR: Fluid-Attenuated Inversion Recovery

**Figure 2:**
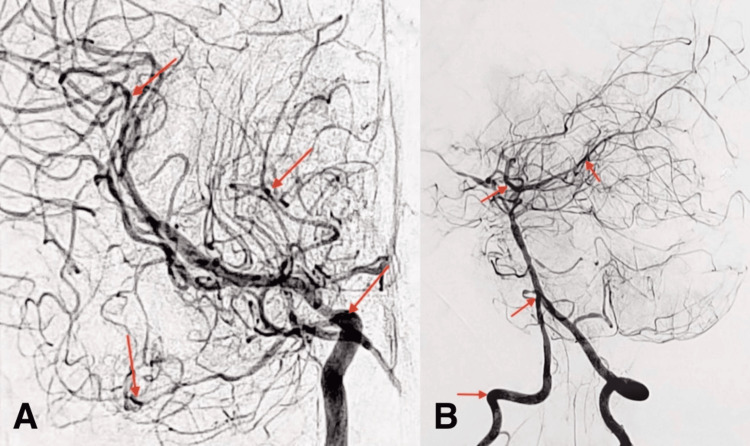
Cerebral arteriography of the patient, showing diffuse vasculitis with multiple multifocal microthrombi. (A) Lateral view of the cerebral arteriography revealing diffuse stenoses and vessel irregularities involving the anterior, middle, and posterior cerebral circulations, including small-caliber distal vessels (red arrows). (B) Frontal view of the arteriography showing multifocal stenoses and vessel irregularities of the anterior and middle cerebral arteries, extending to smaller distal branches (red arrows).

Computed tomography of the chest, abdomen, and pelvis showed hepatosplenomegaly without lymphadenopathy. Brain magnetic resonance imaging (MRI) with angiography identified a right cerebellar ischemic stroke with segmental stenosis of the arterial branches of the Willis polygon (Figure 1). Cerebral arteriography confirmed diffuse cerebral vasculitis with microthrombi, supporting a TMA involving the central nervous system (Figure 2).

**Figure 3:**
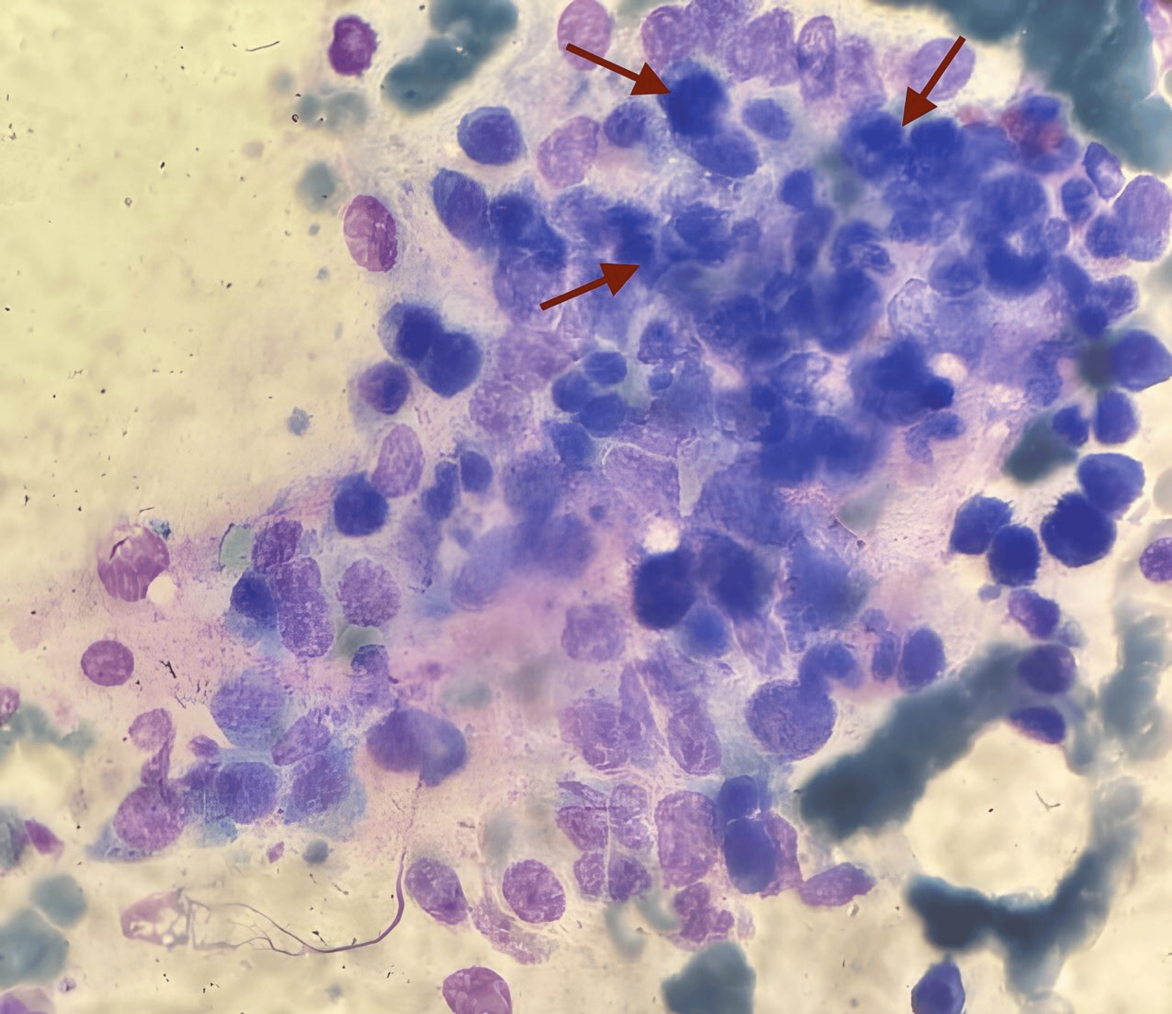
Bone marrow blood smear (May-Grünwald-Giemsa stain) from the present patient, showing histiocytes with hemophagocytosis (red arrows), consistent with hemophagocytic lymphohistiocytosis (HLH).

An exhaustive etiological workup was initiated. ADAMTS13 activity was within the normal range, ruling out thrombotic thrombocytopenic purpura (TTP), while stool cultures and Shiga toxin assays were negative, excluding typical hemolytic uremic syndrome (HUS). Bacterial cultures - including blood, urine, and protected distal respiratory sampling - yielded no growth, and screening for hepatitis B, hepatitis C, HIV, and syphilis was also negative. Autoimmune serologies, including ANA, ANCA, anti-dsDNA, and antiphospholipid antibodies, were unremarkable, and vitamin B12, folate, and complement levels (C3 and C4) were within reference ranges. Transthoracic echocardiography revealed no evidence of infectious endocarditis. A comprehensive metabolic and toxicology screen - including heavy metals, ethanol, and illicit drugs - was unrevealing. Bone marrow aspirate and biopsy demonstrated normocellular marrow with trilineage hematopoiesis and no dysplasia or malignancy, but showed images of hemophagocytosis (Figure 3). A renal or liver biopsy could not be performed due to severe thrombocytopenia and the increased risk of bleeding. Screening for paroxysmal nocturnal hemoglobinuria (PNH) by flow cytometry was negative. Flow cytometry, however, revealed an inverted CD4+/CD8+ ratio, suggesting T-cell activation, which prompted viral investigations. Real-time polymerase chain reaction (PCR) of blood detected CMV DNA at 2.84 log IU/mL and EBV DNA at 3.31 log IU/mL, whereas human herpesvirus (HHV)-6, -7, and -8 PCRs were negative.

Given the presence of persistent high-grade fever; cytopenias (Hb: 5.3 g/dL and platelets: 15 × 10⁹/L); liver dysfunction (AST: 203 IU/L and ALT: 125 IU/L); hepatosplenomegaly; hyperferritinemia (ferritin: 9421 ng/mL); hypertriglyceridemia (triglycerides: 3.1 g/L); hemophagocytosis on bone marrow examination; and evidence of immune activation in the context of viral reactivation, the diagnosis of secondary HLH was also considered. The patient’s HScore was estimated at 259, corresponding to a greater than 99% probability of HLH.

Treatment was promptly initiated with prednisone at 1 mg/kg/day to manage potential immune-mediated endothelial injury, along with piperacillin/tazobactam - adjusted for renal function - to address a possible nosocomial bacterial infection. Intravenous ganciclovir was administered to target CMV and EBV replication, and therapeutic low molecular weight heparin was introduced for confirmed cerebral TMA, with dosing adapted to the platelet count evolution. Supportive therapy included fresh frozen plasma infusions, with rituximab (375 mg/m² weekly) and cyclophosphamide (600 mg/m² monthly) as adjunctive immunosuppressants.

The patient’s hematologic parameters gradually improved: platelet count and hemoglobin increased, schistocytes disappeared, and renal and hepatic functions recovered. Neurologic status improved following 15 days of intubation, with support from motor and respiratory rehabilitation.

After 25 days in the ICU, she was successfully weaned off ventilation and transferred back to the hematology ward, in a stable condition, for further monitoring and tapering of immunosuppression.

## Discussion

This case highlights a rare and severe presentation of TMA and secondary HLH, both triggered by co-infection with CMV and EBV in an immunocompetent young adult, leading to multiorgan failure and severe central nervous system involvement.

Secondary HLH is a life-threatening hyperinflammatory condition, characterized by the proliferation and accumulation of macrophage-like histiocytes and uncontrolled immune activation, leading to a cytokine storm responsible for tissue damage and multiorgan dysfunction. It can be precipitated by various triggers, notably viral infections, hematologic malignancies, and autoimmune diseases [1]. In this patient, the diagnosis was based on the HLH-2004 criteria and an HScore [5] of 259, indicating >99% probability of HLH. The presence of persistent high-grade fever, bicytopenia, hepatosplenomegaly, hyperferritinemia, hypertriglyceridemia, hemophagocytosis, and liver dysfunction met six out of the eight HLH-2004 diagnostic criteria.

In parallel, the patient developed a severe MAHA with neurologic involvement, evidenced by diffuse cerebral vasculitis on imaging. The exclusion of alternative etiologies - normal ADAMTS13 activity, absence of Shiga toxin, negative autoimmune and malignancy workups - and active EBV and CMV viremia strongly supported a diagnosis of virus-induced systemic endothelial injury overlapping with HLH.

CMV and EBV are lymphotropic herpesviruses capable of establishing latency and inducing immune dysregulation. While typically affecting immunocompromised hosts, severe manifestations in immunocompetent individuals have been documented in rare cases [6,7]. Both viruses are known to induce massive cytokine release (IL-6, IFN-γ, TNF-α) and have been implicated in endothelial injury, immune activation, and HLH pathogenesis [8,9]. In particular, EBV-driven T-cell activation is a classic driver of HLH8, whereas CMV has been shown to directly infect endothelial cells and promote prothrombotic states [10,11]. Additionally, immune complex deposition and molecular mimicry may lead to the development of autoreactive antibodies targeting endothelial cells [12]. EBV has also been associated with vasculitis-like syndromes and, in rare cases, central nervous system arterial thrombosis [13-15]. Co-infection with EBV and CMV may synergistically exacerbate viral replication and immune activation, thereby increasing the risk of HLH and TMA. This underscores a pathophysiological continuum in which CMV and EBV co-infection serves as a dual trigger for both hyperinflammatory immune activation (HLH) and vascular endothelial injury (TMA). Their concurrent occurrence suggests a shared upstream mechanism of virus-driven immune dysregulation. What makes this case particularly noteworthy is the absence of any underlying immunodeficiency.

Management in such cases is not standardized and remains based on pathophysiological reasoning and case-based evidence. In our patient, a multimodal therapeutic approach was adopted, including antiviral therapy (ganciclovir), high-dose corticosteroids, immunosuppressive agents (rituximab and cyclophosphamide), therapeutic anticoagulation, fresh frozen plasma, and supportive care. Rituximab and cyclophosphamide were used for a dual purpose: to provide immunosuppressive therapy aimed at controlling the HLH, and to target the endothelial activation potentially responsible for the TMA. Their use is supported in other immune-mediated TMAs, HLH, and autoimmune-associated hemophagocytic syndrome (AAHS) [2,16-19].

## Conclusions

This is a rare clinical case of concurrent HLH and TMA in a young, immunocompetent patient, highlighting an unusual and severe manifestation of virus-induced immune dysregulation. It expands the current understanding of infection-related HLH and TMAs, and emphasizes the need to remain vigilant for viral triggers, even in individuals without underlying immunodeficiency. Early recognition and prompt initiation of appropriate therapy are critical to improve outcomes, given the high risk of rapid deterioration. The combination of treatment and supportive measures undertaken in this case reflects the need for an individualized and multimodal approach. This observation illustrates the value of maintaining a broad differential diagnosis and multidisciplinary collaboration when facing complex presentations. Additional reports of similar cases will be essential to improve awareness and to guide future diagnostic and therapeutic strategies.
